# Significance of urinary C-megalin excretion in vitamin D metabolism in pre-dialysis CKD patients

**DOI:** 10.1038/s41598-019-38613-8

**Published:** 2019-02-18

**Authors:** Norikazu Toi, Masaaki Inaba, Eiji Ishimura, Naoko Tsugawa, Yasuo Imanishi, Masanori Emoto, Yoshiaki Hirayama, Shinya Nakatani, Akihiko Saito, Shinsuke Yamada

**Affiliations:** 1 Department of Metabolism, Endocrinology, and Molecular Medicine, Osaka City University Graduate School of Medicine, Osaka, Japan; 2 Department of Nephrology, Osaka City University Graduate School of Medicine, Osaka, Japan; 3 Department of Nephrology, Meijibashi Hospital, Osaka, Japan; 4 Department of Health and Nutrition, Osaka Shoin Women’s University, Osaka, Japan; 5 Reagent Research and Development Department, Denka Co, Ltd., Gosen, Japan; 6 Department of Applied Molecular Medicine, Kidney Research Center, Niigata University Graduate School of Medical and Dental Sciences, Niigata, Japan

## Abstract

Serum 1,25(OH)_2_D and 24,25(OH)_2_D are decreased in CKD. Megalin in proximal tubular epithelial cells reabsorbs glomerular-filtered 25(OH)D-DBP complex to convert 25(OH)D to 1,25(OH)_2_D and 24,25(OH)_2_D. Urinary C-megalin excretion is increased *via* exocytosis from injured nephrons overloaded with megalin-mediated protein metabolism. This study investigated the significance of urinary C-megalin excretion in vitamin D metabolism in 153 pre-dialysis CKD patients. Urinary C-megalin was positively associated with urinary protein, β_2_MG and α_1_MG, and exhibited negative correlations with serum 25(OH)D, 1,25(OH)_2_D and 24,25(OH)_2_D. Multiple regression analysis showed that urinary C-megalin had a significantly negative association with 25(OH)D. Serum 1,25(OH)_2_D and 24,25(OH)_2_D, as well as 1,25(OH)_2_D/25(OH)D and 24,25(OH)_2_D/25(OH)D ratios, showed positive correlations with eGFR. Additionally, wholePTH was positively associated with 1,25(OH)_2_D/25(OH)D and 1,25(OH)_2_D/24,25(OH)_2_D, while FGF23 was positively associated with 24,25(OH)_2_D/25(OH)D and negatively with 1,25(OH)_2_D/24,25(OH)_2_D. Urinary C-megalin emerged as an independent factor positively associated with 1,25(OH)_2_D/25(OH)D and 1,25(OH)_2_D/24,25(OH)_2_D. Although 1,25(OH)_2_D and 24,25(OH)_2_D are decreased in CKD patient serum, our findings suggest that PTH and FGF23 retain their effects to regulate vitamin D metabolism even in the kidneys of these patients, while production of 1,25(OH)_2_D and 24,25(OH)_2_D from 25(OH)D is restricted due to either impairment of megalin-mediated reabsorption of the 25(OH)D-DBP complex or reduced renal mass.

## Introduction

Previous results including ours have demonstrated that 1,25-dihydroxyvitamin D [1,25(OH)_2_D] and 24,25-dihydroxyvitamin D [24,25(OH)_2_D] in serum become progressively decreased in pre-dialysis patients with chronic kidney disease (CKD), as estimated creatinine clearance is lower than 50 mL/min, even though serum 25-hydroxyvitamin D [25(OH)D], a precursor of 1,25(OH)_2_D and 24,25(OH)_2_D, remains unchanged^1^. Since metabolism of 25(OH)D to 1,25(OH)_2_D and 24,25(OH)_2_D occurs in proximal tubular epithelial cells (PTECs)^2^, dysfunction of this nephron site due to deterioration caused by CKD is likely responsible for reduced serum levels of 1,25(OH)_2_D and 24,25(OH)_2_D in affected patients^3^. Vitamin D, a prohormone that binds to vitamin D-binding protein (DBP) in circulation, is metabolically converted to 25(OH)D in the liver, and then to 1,25(OH)_2_D or 24,25(OH)_2_D in the kidneys. It has been reported that most 25(OH)D in circulation is bound to DBP, although it also exists in albumin- and lipoprotein-bound forms and also exist in the form of free 25(OH)D, while 25(OH)D-DBP complexes are continuously filtered across the glomerular filtration barrier^3^ and taken up *via* megalin by PTECs^4,5^. Megalin, a 600-kDa glycoprotein and member of the low-density lipoprotein receptor family, is internalized by endocytosis to form endocytic vesicles and then recycled to the plasma membrane^5,6^. Endocytic ligands of megalin, including 25(OH)D-DBP complexes, are trafficked to lysosomes for degradation, during which 25(OH)D escapes from the pathway and is transported to mitochondria for conversion to 1,25(OH)_2_D or 24,25(OH)_2_D^5–7^.

Megalin exists in urine in both ectodomain (A-megalin) and full-length (C-megalin) forms, which can be measured using amino- and carboxyl-terminal enzyme-linked immunosorbent assay (ELISA) results, respectively^8^. Using those assays, Saito and colleagues found that urinary C-megalin is increased along with the progression of diabetic kidney disease^8^ and IgA nephropathy^9^. Also, De *et al*. recently showed that urinary C-megalin excretion is increased via exocytosis from PTECs injured by endo-lysosomal overload caused by megalin-mediated protein metabolism^10^. The clinical significance of urinary C-megalin, which represents “metabolic nephron load,” was confirmed by results showing that it is also a good marker to diagnose pediatric patients with renal scarring, which often occurs following a febrile urinary infection and is often accompanied by residual nephron overload^11^. Therefore, urinary C-megalin excretion likely reflects phenotypic changes that progress in PTECs in CKD patients^12^. The expression and function of megalin in PTECs are considered to be affected by progression of CKD^13,14^. Therefore, increased exosome megalin loss may be associated with a decrease in its renal expression and altered function for vitamin D handing, providing insight into the mechanisms underlying vitamin D metabolism in affected patients.

With that background in mind, the aim of the present study was to determine the significance of urinary C-megalin excretion in vitamin D metabolism in pre-dialysis CKD patients.

## Results

### Clinical characteristics of CKD patients

The clinical characteristics of the 153 CKD patients enrolled in the present study are shown in Table 1. Values for median eGFR, corrected calcium (cCa), and phosphate (Pi) were 30.0 (5.0–58.8) mL/min/1.73 m^2^, 9.4 (6.7–10.7) mg/dL, and 3.6 (2.0–7.2) mg/dL, respectively, while those for whole parathyroid hormone (wholePTH) and fibroblast growth factor (FGF) 23 in serum were 49.4 (6.8–645.0) pg/mL and 77.5 (10.0–840.0) pg/mL, respectively, well above their respective normal upper limits^15,16^. Additionally, urinary C-megalin was 0.6 (0–8.5) pmol/g Creatinine (Cr), higher than the reported value for healthy individuals (0.15 pmol/g Cr, n = 160)^8,9^.

**Table 1:** Clinical characteristics.

No. of patients	153
Age, years (range)	67 (26–90)
Gender, male/female	101/52
BMI, kg/m^2^	23.1 ± 4.2
T2DM, no. (%)	48 (31.3)
HbA1c (NGSP), %	5.8 ± 0.7
Alb, g/dL	3.8 ± 0.5
eGFR, mL/min/1.73 m^2^ (range)	30.0 (5.0–58.8)
AST, IU/L	21.5 ± 12.1
ALT, IU/L	20.5 ± 23.1
cCa, mg/dL (range)	9.4 (6.7–10.7)
Pi, mg/dL (range)	3.6 (2.0–7.2)
wholePTH, pg/mL (range)	49.4 (6.8–645)
FGF23, pg/mL (range)	77.5 (10.0–840)
25(OH)D, ng/mL (range)	14.2 (2.6–42.3)
24,25(OH)_2_D, ng/mL (range)	0.5 (0–2.5)
1,25(OH)_2_D, pg/mL (range)	34.8 (5.1–88.3)
Urinary C-megalin/Cr, pmol/gCr (range)	0.6 (0–8.5)
Urinary protein/Cr, g/gCr (range)	1.5 (0–15.0)
Urinary β_2_MG/Cr, μg/gCr (range)	1790.0 (18.0–93701)
Urinary α_1_MG/Cr, mg/gCr (range)	32.2 (2.6–1129)

Values are expressed as the mean ± SD.

Median values (range) are shown for variables with skewed distributions.

BMI = body mass index, DM = diabetes mellitus, HbA1c = hemoglobin A1c, Alb = albumin, eGFR = estimated glomerular filtration rate, AST = aspartate aminotransferase, ALT = alanine transaminase, cCa = corrected calcium, Pi = phosphate, PTH = parathyroid hormone, FGF23 = fibroblast growth factor 23, 25(OH)D = 25-hydroxyvitamin D, 24,25(OH)_2_D = 24,25-dihydroxyvitamin D, 1,25(OH)_2_D = 1,25-dihydroxyvitamin D, MG = microglobulin.

### Relationship of urinary excreted β_2_-microglobulin (β_2_MG) and α_1_-microglobulin (α_1_MG) with urinary C-megalin excretion in CKD patients

**Figure 1:**
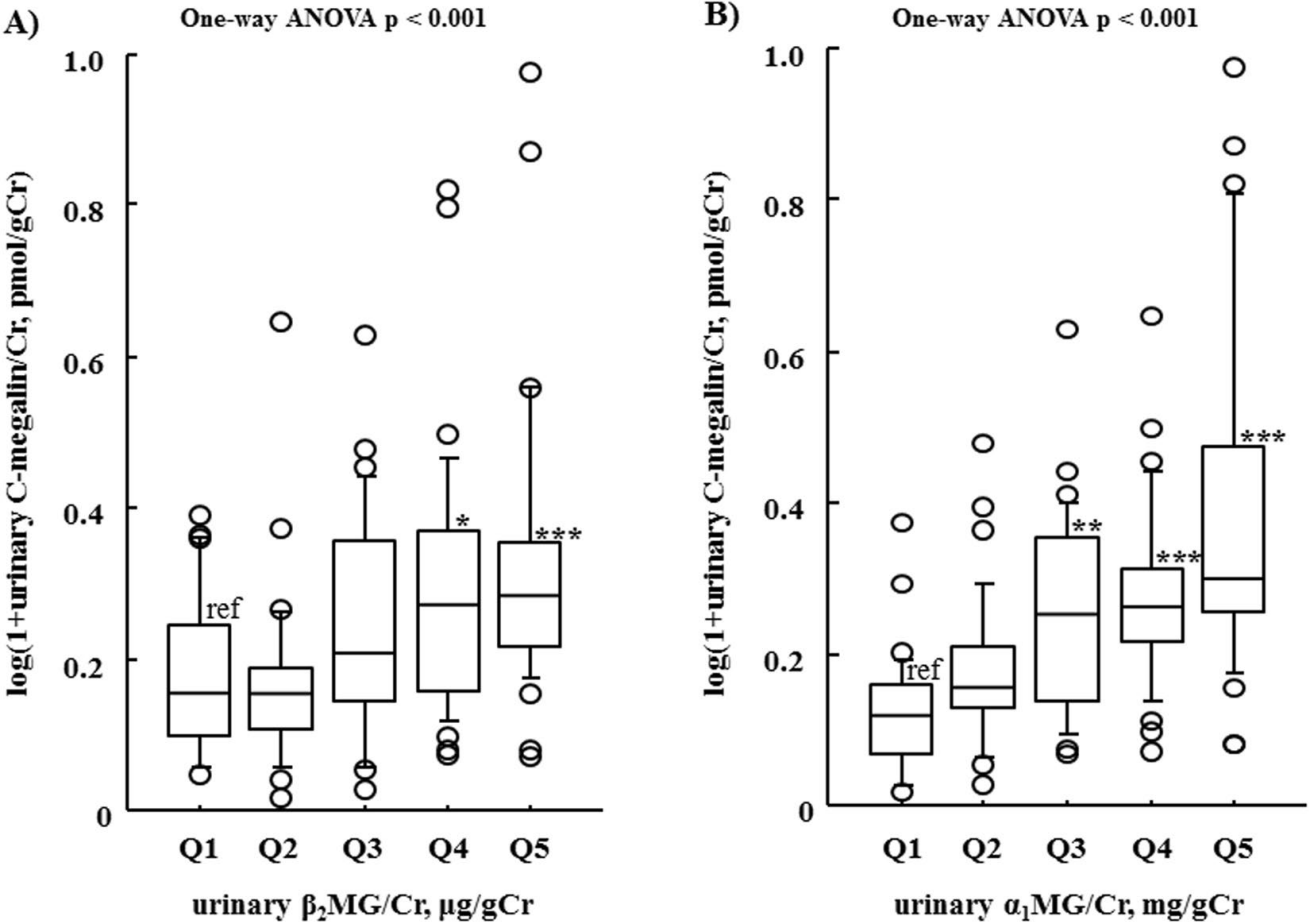
Relationship of urinary C-megalin excretion with urinary excretion of β_2_MG and α_1_MG, after stratification of those into quintiles. Mean and IQR values for urinary C-megalin excretion are shown for the β_2_MG and α_1_MG quintiles. Urinary C-megalin excretion was significantly increased in the higher urinary β_2_MG and α_1_MG quintiles. The quintile values for urinary β_2_MG (μg/g Cr) were as follows: Q1, 0.0 to 265.0 (n = 30); Q2, 265.1 to 963.2 (n = 31); Q3, 963.3 to 3302.7 (n = 31); Q4, 3302.8 to 21536.4 (n = 31); and Q5, ≥21536.5 (n = 30), while those for urinary α_1_MG (mg/g Cr) were 0.0 to 15.2 (n = 30); 15.3 to 26.0 (n = 31); 26.1 to 49.2 (n = 31); 49.3 to 92.4 (n = 31); and ≥92.5 (n = 30), respectively. Differences between quintiles were examined by one-way ANOVA. *P < 0.05, **P < 0.01, ***P < 0.001, as compared with Q1 using Dunnett’s test.

To examine whether urinary C-megalin excretion might be a clinically relevant assay factor for PTECs injury, relationships between urinary C-megalin excretion with urinary of β_2_MG and α_1_MG, which are established markers for PTECs dysfunction and endocytic ligands of megalin in PTECs^14^, were investigated. We compared those markers after stratification of β_2_MG/Cr and α_1_MG/Cr in 153 CKD patients by quintiles (Fig. 1). Urinary C-megalin excretion became significantly greater in quintiles with higher levels of either urinary excretion of β_2_MG/Cr (p < 0.001) or α_1_MG/Cr (p < 0.001), indicating that urinary C-megalin excretion is a relevant marker for PTECs injury and linked with the endocytic function of megalin in CKD patients.

### Univariate and multivariate analyses of associations of various clinical variables with urinary C-megalin in CKD patients

Among the various clinical variables subjected to univariate analysis of correlation with urinary C-megalin excretion in the present CKD patients, serum levels of wholePTH (ρ = 0.224, p = 0.007) and FGF23 (ρ = 0.280, p < 0.001), and urinary levels of protein/Cr (ρ = 0.603, p < 0.001), β_2_MG/Cr (ρ = 0.415, p < 0.001), and α_1_MG/Cr (ρ = 0.594, p < 0.001) showed significantly positive correlations with urinary C-megalin excretion, whereas Body Mass Index (BMI) (ρ = -0.186, p = 0.033), serum albumin (ρ = -0.391, p < 0.001), and eGFR (ρ = -0.389, p < 0.001) had significantly negative correlations (data not shown). These findings were then further examined using multiple regression analysis (Table 2). When age, gender, BMI, HbA1c, albumin, log(eGFR), log(wholePTH), log(FGF23), and log(1 + urinary protein/Cr) were included as independent variables (model 1), log(1 + urinary protein/Cr) emerged as significant independent factors positively associated with urinary C-megalin. When urinary log(1 + urinary protein/Cr) was replaced with log(urinary β_2_MG/Cr) (model 2) or log(urinary α_1_MG/Cr) (model 3), those factors also showed a significant and independent association with urinary C-megalin. Analyses with models 4 and 5, which included urinary log(1 + urinary protein/Cr) together with log(urinary β_2_MG/Cr) or log(urinary α_1_MG/Cr), respectively, each demonstrated urinary log(1 + urinary protein/Cr) as a significant independent factor associated with urinary C-megalin. Notably, neither log(wholePTH) nor log(FGF23) was found to be associated with urinary C-megalin.

**Table 2:** Multiple regression analysis of association with log(1 + urinary C-megalin/Cr).

	Model 1		Model 2		Model 3		Model 4		Model 5	
	β	p	β	p	β	p	β	p	β	p
Age	0.014	0.876	− 0.014	0.872	−0.052	0.555	0.013	0.883	−0.015	0.865
Gender, male/female 1/2	−0.077	0.341	−0.065	0.446	−0.077	0.349	−0.065	0.424	−0.074	0.359
BMI	−0.019	0.831	−0.058	0.519	−0.019	0.829	−0.008	0.924	0.003	0.972
HbA1c	−0.030	0.719	−0.019	0.823	−0.052	0.539	−0.033	0.689	−0.049	0.553
Alb	−0.250	0.009*	−0.445	<0.001*	−0.397	<0.001*	−0.269	0.006*	−0.274	0.004*
log(eGFR)	−0.184	0.206	−0.158	0.346	−0.058	0.720	−0.094	0.558	−0.060	0.704
log(wholePTH)	0.016	0.885	0.043	0.715	−0.027	0.815	0.020	0.859	−0.017	0.881
log(FGF23)	−0.083	0.465	−0.015	0.902	−0.005	0.965	−0.047	0.686	−0.039	0.733
log(1 + urinary protein/Cr)	0.400	<0.001*	—	—	—	—	0.363	<0.001*	0.293	0.013*
log(urinary β_2_MG/Cr)	—	—	0.223	0.027*	—	—	0.128	0.199	—	—
log(urinary α_1_MG/Cr)	—	—	—	—	0.395	<0.001*	—	—	0.232	0.070
R^2^ (p)	0.392 (<0.001)		0.336 (<0.001)		0.376 (<0.001)		0.402 (<0.001)		0.410 (<0.001)	

β is the standardized regression coefficient. *p < 0.05.

BMI = body mass index, HbA1c = hemoglobin A1c, Alb = albumin, eGFR = estimated glomerular filtration rate, PTH = parathyroid hormone, FGF23 = fibroblast growth factor 23, MG = microglobulin.

### Correlations of serum vitamin D metabolites with eGFR in CKD patients

**Figure 2:**
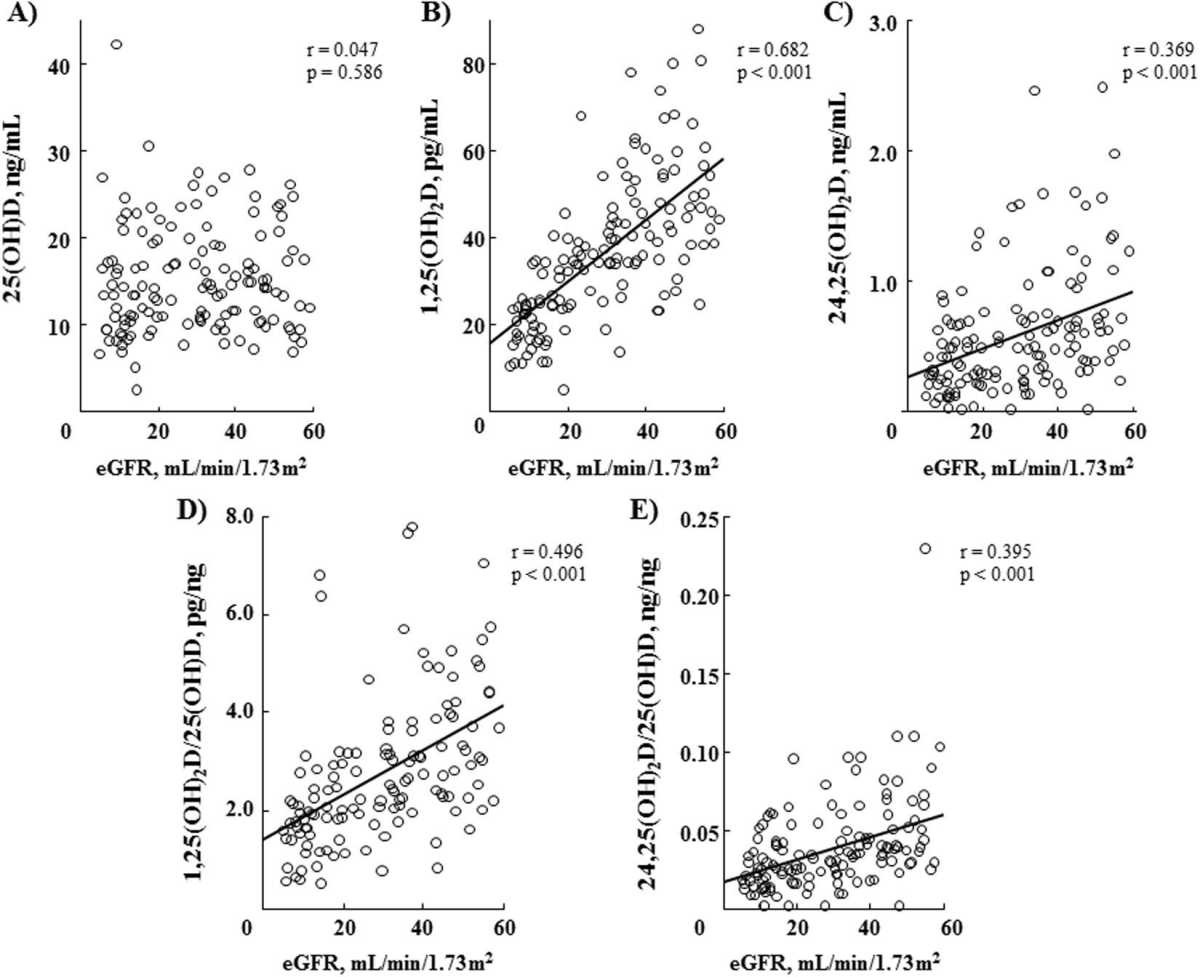
Correlations between eGFR and vitamin D metabolites. eGFR was significantly correlated in a positive manner with serum 1,25(OH)_2_D (**B**: r = 0.682, p < 0.001) and 24,25(OH)_2_D (**C**: r = 0.369, p < 0.001), but not with serum 25(OH)D (**A**: r = 0.047, p = 0.586), and also in a positive manner with serum 1,25(OH)_2_D/25(OH)D ratio (**D**: r = 0.496, p < 0.001) and 24,25(OH)_2_D/25(OH)D ratio (**E**: r = 0.395, p < 0.001).

As we previously reported^1^, in the present 153 CKD patients, there was a significantly positive correlation of eGFR with serum 1,25(OH)_2_D (r = 0.682, p < 0.001) and 24,25(OH)_2_D (r = 0.369, p < 0.001), but not with serum 25(OH)D (r = 0.047, p = 0.586) (Fig. 2A–C). The correlation of serum 1,25(OH)_2_D with eGFR was stronger than that of 24,25(OH)_2_D (p < 0.001). Additionally, serum 1,25(OH)_2_D/25(OH)D ratio (r = 0.496, p < 0.001) and 24,25(OH)_2_D/25(OH)D ratio (r = 0.395, p < 0.001) showed significantly positive correlations with eGFR in these patients (Fig. 2D,E).

### Correlations of serum vitamin D metabolites with urinary C-megalin excretion in CKD patients

**Figure 3:**
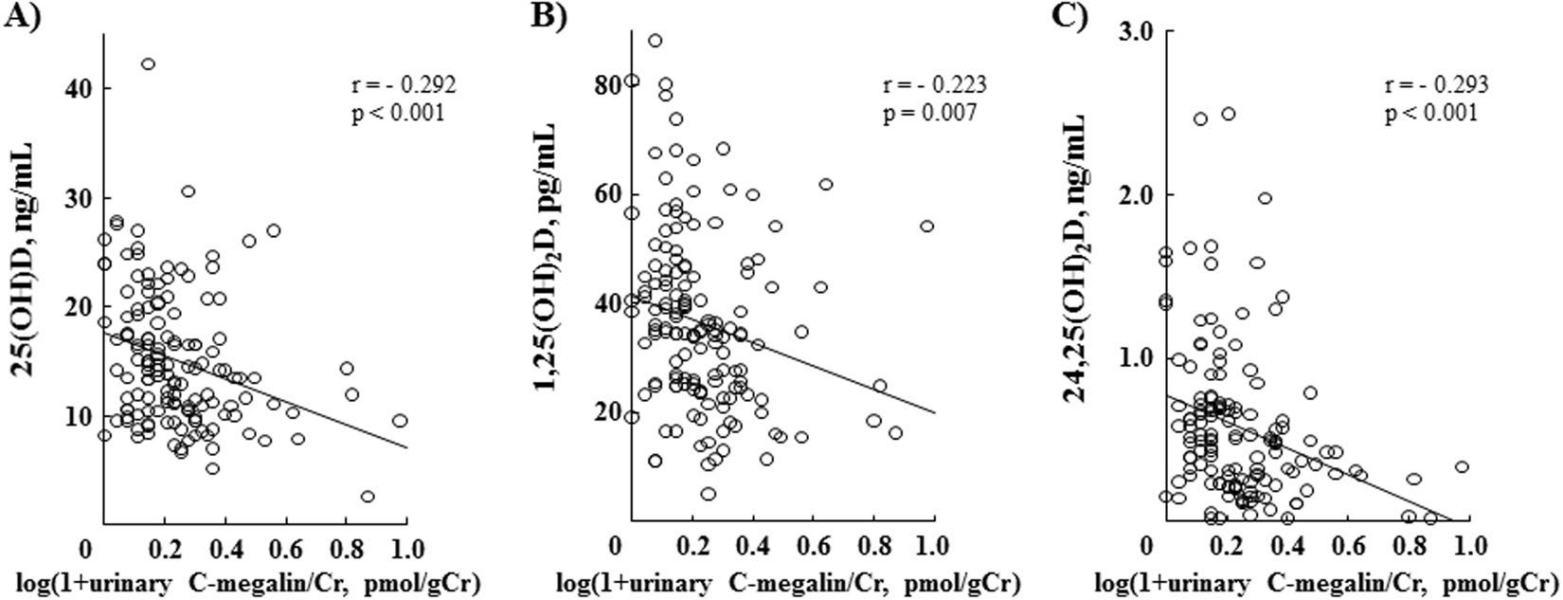
Correlations between urinary C-megalin and 3 vitamin D metabolites. Urinary C-megalin excretion exhibited a significantly negative correlation with 25(OH)D (**A**: r = −0.292, p < 0.001), 1,25(OH)_2_D (**B**: r = −0.223, p = 0.007) and 24,25(OH)_2_D (**C**: r = − 0.293, p < 0.001).

We next examined the correlation of urinary C-megalin excretion with the serum vitamin D metabolites 25(OH)D (Fig. 3A), 1,25(OH)_2_D (Fig. 3B), and 24,25(OH)_2_D (Fig. 3C). Those results showed that each was significantly correlated in a negative manner (r = −0.292, p < 0.001, r = −0.223, p = 0.007 and r = −0.293, p < 0.001, respectively) with urinary C-megalin excretion in the present 153 CKD patients. These findings indicate that urinary loss of megalin via exosomes is associated with reduced serum levels of vitamin D metabolites in CKD.

### Univariate and multivariate analyses of associations of various clinical variables with serum 25(OH)D in CKD patients

We also examined univariate analysis to determine the correlations of various clinical variables with serum 25(OH)D in our CKD patients. Serum albumin (ρ = 0.306, p < 0.001) showed a significantly positive correlation with serum 25(OH)D, whereas female gender (ρ = −0.195, p = 0.021), serum wholePTH (ρ = −0.190, p = 0.028), and urinary levels of C-megalin/Cr (ρ = −0.310, p < 0.001), protein/Cr (ρ = −0.318, p < 0.001), β_2_MG/Cr (ρ = −0.204, p = 0.016), and α_1_MG/Cr (ρ = −0.254, p = 0.003) showed significantly negative correlations with serum 25(OH)D (data not shown). Those correlations were further examined using multiple regression analysis (Table 3). When age, gender, BMI, HbA1c, albumin, log(eGFR), log(wholePTH), log(FGF23), and log(1 + urinary C-megalin/Cr) were included as independent variables (model 1), log(1 + urinary C-megalin/Cr) in addition to female gender, and log(wholePTH) emerged as significant independent factors negatively associated with serum 25(OH)D. When urinary log(1 + urinary C-megalin/Cr) was replaced with urinary log(1 + urinary protein/Cr) (model 2), log(1 + urinary protein/Cr) was shown to have a significantly negative association with serum 25(OH)D. In analysis with model 3, which simultaneously included urinary log(1 + urinary C-megalin/Cr) and log(1 + urinary protein/Cr), log(1 + urinary C-megalin/Cr), but not log(1 + urinary protein/Cr), retained a significantly negative association with serum 25(OH)D. It is also important to note that serum wholePTH correlated significantly in a negative manner with 25(OH)D (ρ = −0.190, p = 0.028), and that multivariate regression analysis showed that serum wholePTH retained a significantly negative association with serum 25(OH)D in models 1, 2, and 3 (Table 3).

**Table 3:** Multivariate regression analysis to elucidate factors associated with log[25(OH)D].

	Model 1		Model 2		Model 3	
	β	p	β	p	β	p
Age	−0.054	0.571	−0.066	0.496	−0.068	0.476
Gender, male/female 1/2	−0.183	0.043*	−0.162	0.074*	−0.184	0.040*
BMI	0.074	0.430	0.045	0.638	0.044	0.636
HbA1c	0.048	0.601	0.064	0.489	0.056	0.535
Alb	0.252	0.013*	0.257	0.014*	0.186	0.079
log(eGFR)	−0.217	0.185	−0.221	0.189	−0.282	0.091
log(wholePTH)	−0.305	0.018*	−0.279	0.032*	−0.294	0.021*
log(FGF23)	0.005	0.970	0.022	0.868	0.004	0.974
log(1 + urinary C-megalin/Cr)	−0.314	0.002*	—	—	−0.250	0.019*
log(1 + urinary protein/Cr)	—	—	−0.300	0.009*	−0.204	0.082
R^2^ (p)	0.325 (<0.001)		0.309 (<0.001)		0.345 (<0.001)	

β is the standardized regression coefficient. *p < 0.05.

25(OH)D = 25-hydroxyvitamin D, BMI = body mass index, HbA1c = hemoglobin A1c, Alb = albumin, eGFR = estimated glomerular filtration rate, PTH = parathyroid hormone, FGF23 = fibroblast growth factor 23.

### Associations of serum vitamin D metabolites and their ratios in serum wholePTH and FGF23 quintiles

**Figure 4:**
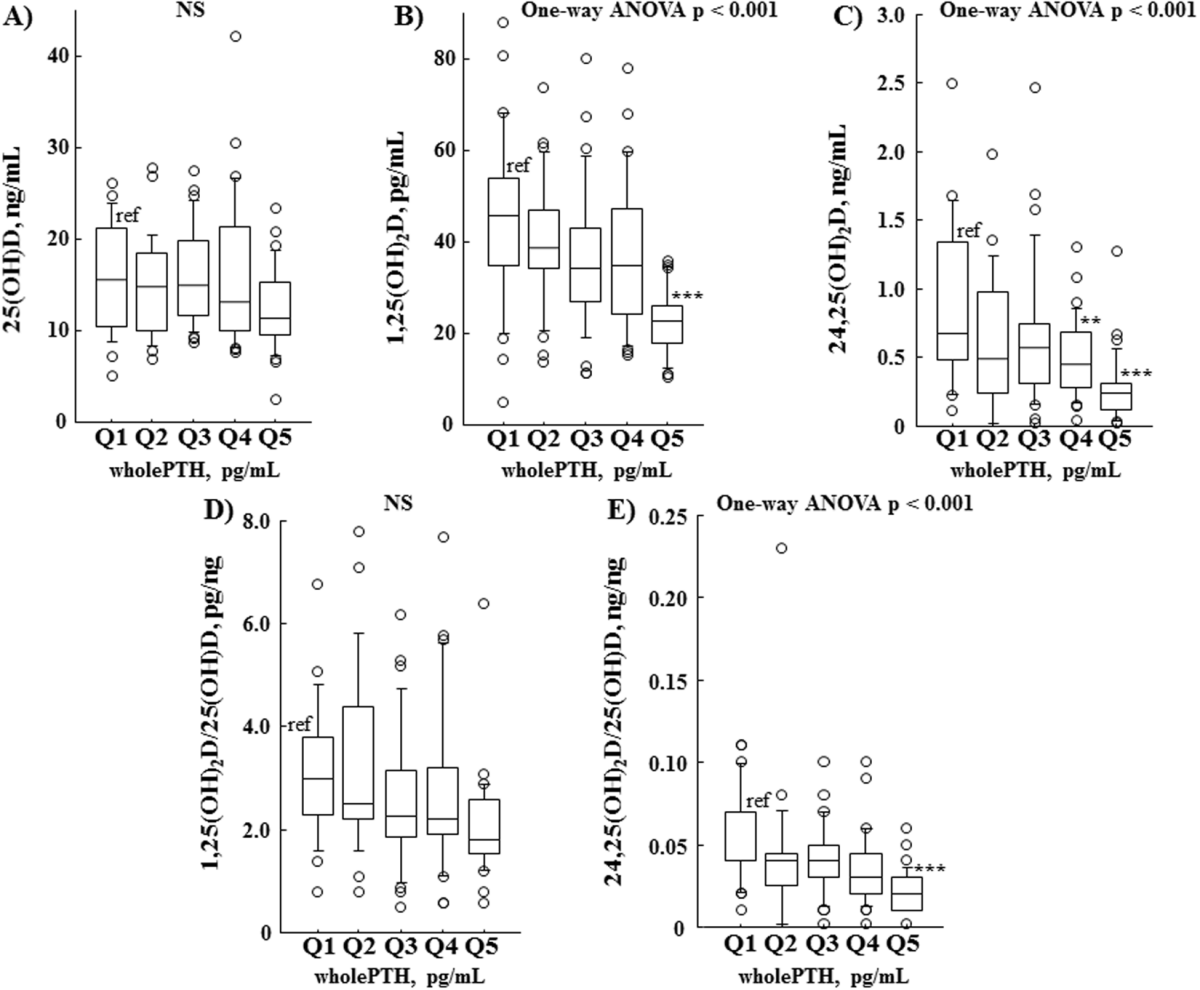
Relationships of serum vitamin D metabolites and their ratios in serum wholePTH quintiles. Serum 1,25(OH)_2_D (**B**) and 24,25(OH)_2_D (**C**) were significantly decreased in the higher wholePTH quintiles, in contrast with an insignificant reduction in serum 25(OH)D (**A**) regardless of wholePTH quintile. Furthermore, the serum 1,25(OH)_2_D/25(OH)D ratio (**D**) was not different among the wholePTH quintiles, while serum 24,25(OH)_2_D/25(OH)D ratio (**E**) was significantly lower in the higher quintiles. Values showing differences for wholePTH (pg/mL) in the quintiles were as follows: Q1, 0.0 to 26.3 (n = 29); Q2, 26.4 to 39.8 (n = 29); Q3, 39.9 to 63.1 (n = 30); Q4, 63.2 to 112.3 (n = 30); and Q5, ≥112.4 (n = 29). Differences between quintiles were examined by one-way ANOVA. *P < 0.05, **P < 0.01, ***P < 0.001, as compared with Q1 using Dunnett’s test.

To determine the significance of the effect of wholePTH on vitamin D metabolism in CKD patients, we examined serum 25(OH)D (Fig. 4A), 1,25(OH)_2_D (Fig. 4B), and 24,25(OH)_2_D (Fig. 4C), as well as 1,25(OH)_2_D/25(OH)D (Fig. 4D) and 24,25(OH)_2_D/25(OH)D (Fig. 4E) ratios after stratification of serum wholePTH into quintiles. Serum wholePTH was increased as eGFR became lower due to development of secondary hyperparathyroidism, thus higher quintiles of serum wholePTH exhibited lower eGFR. Our results showed that serum 1,25(OH)_2_D and 24,25(OH)_2_D were significantly decreased in the higher wholePTH quintiles in contrast with an insignificant reduction in serum 25(OH)D in the wholePTH quintiles. On the other hand, the serum 1,25(OH)_2_D/25(OH)D ratio did not change in the wholePTH quintiles, though the serum 24,25(OH)_2_D/25(OH)D ratio became significantly lower in the higher quintiles.

**Figure 5:**
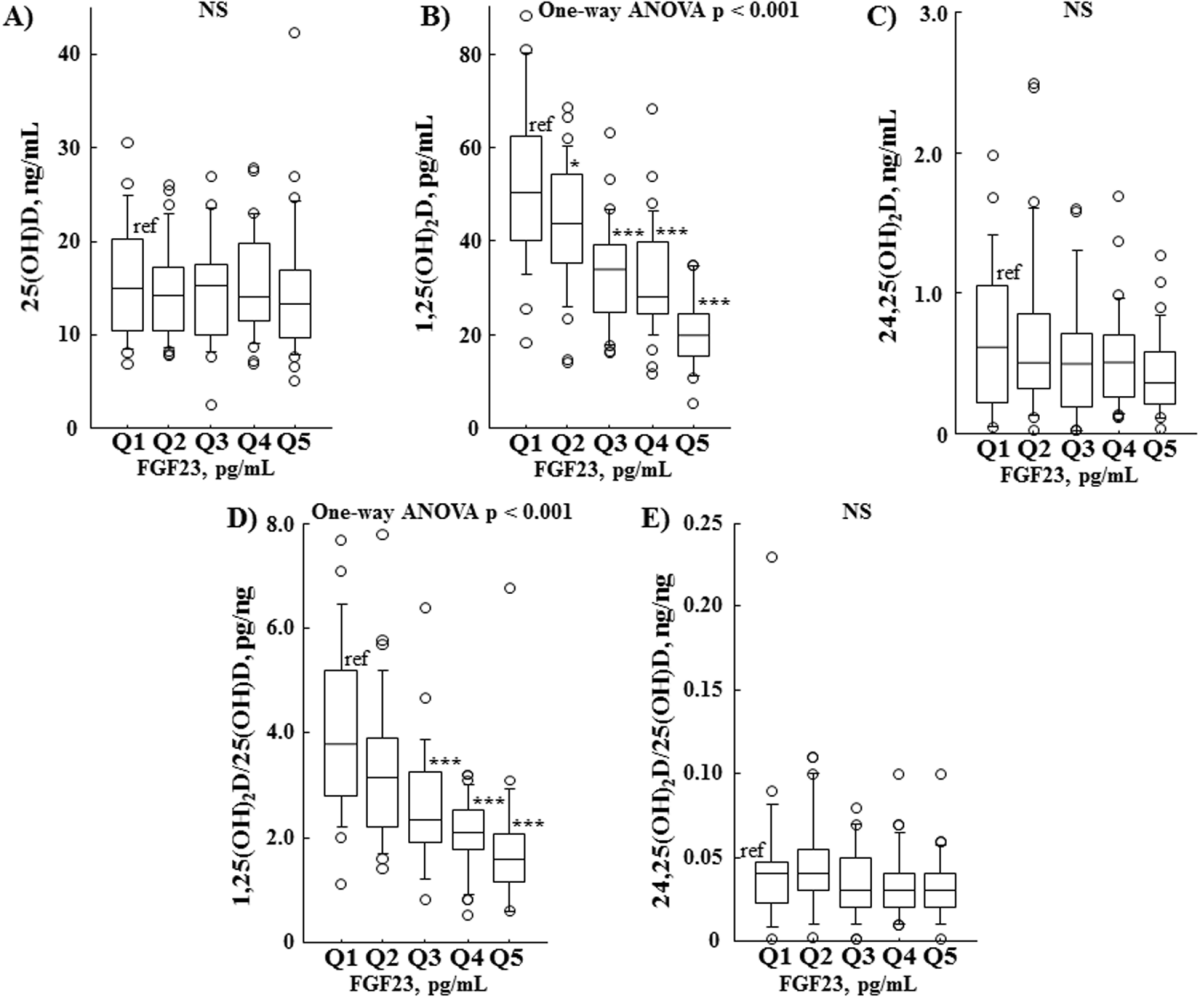
Relationship of serum vitamin D metabolites and their ratios in serum FGF23 quintiles. Serum 1,25(OH)_2_D (**B**) was significantly decreased in the higher FGF23 quintiles in contrast with insignificant reductions in serum 25(OH)D (**A**) and 24,25(OH)_2_D (**C**) regardless of FGF23 quintile. Furthermore, the serum 24,25(OH)_2_D/25(OH)D ratio (**E**) was not different among the FGF23 quintiles, while serum 1,25(OH)_2_D/25(OH)D ratio (**D**) was significantly lower in the higher FGF23 quintiles. Values showing differences for FGF23 in the quintiles were as follows: Q1, 0.0 to 48.9 (n = 26); Q2, 49.0 to 68.5 (n = 33); Q3, 68.6 to 93.9 (n = 28); Q4, 94.0 to 157.3 (n = 33); and Q5, ≥ 157.4 (n = 30). Differences between quintiles were examined by one-way ANOVA. *P < 0.05, **P < 0.01, ***P < 0.001, as compared with Q1 using Dunnett’s test.

Similarly, higher serum FGF23 quintiles exhibited lower levels of eGFR. Serum 1,25(OH)_2_D (Fig. 5B) was significantly decreased in those quintiles in contrast with insignificant reductions in serum 25(OH)D (Fig. 5A) and 24,25(OH)_2_D (Fig. 5C) in all of the FGF23 quintiles. Accordingly, serum 24,25(OH)_2_D/25(OH)D ratio (Fig. 5E) was not different in the FGF23 quintiles, while the serum 1,25(OH)_2_D/25(OH)D ratio (Fig. 5D) was significantly lower.

### Multivariate regression analysis of various clinical variables associated with serum log[1,25(OH)_2_D/25(OH)D], log[24,25(OH)_2_D/25(OH)D], and log[1,25(OH)_2_D/24,25(OH)_2_D] in CKD patients

Conversion of 25(OH)D to 1,25(OH)_2_D, an activation step of 25(OH)D, and to 24,25(OH)_2_D, an inactivation step of 25(OH)D, has been reported to be mediated by 25(OH)D 1α-hydroxylase [1α(OH)ase] (CYP27B1) and 25(OH)D 24-hydroxylase [24(OH)ase] (CYP24A1), respectively^17,18^. Furthermore, accumulating evidence indicates that 1,25(OH)_2_D/25(OH)D and 24,25(OH)_2_D/25(OH)D ratios are clinically relevant for renal 1α(OH)ase and 24(OH)ase, respectively^18,19^, while 1,25(OH)_2_D/24,25(OH)_2_D ratio has been shown to be a valid measurement for 25(OH)D activation^19^. In this study, we performed multivariate regression analysis to elucidate clinical variables associated with serum log[1,25(OH)_2_D/25(OH)D], log[24,25(OH)_2_D/25(OH)D], and log[1,25(OH)_2_D/24,25(OH)_2_D] in CKD patients (Table 4). When age, gender, BMI, HbA1c, serum albumin, log(eGFR), log(wholePTH), log(FGF23), and log(1 + urinary C-megalin/Cr) were included as independent variables, log(eGFR) was significantly and positively associated with serum log[1,25(OH)_2_D/25(OH)D] and log[24,25(OH)_2_D/25(OH)D]. Furthermore, log(wholePTH) was positively associated with log[1,25(OH)_2_D/25(OH)D] and log[1,25(OH)_2_D/24,25(OH)_2_D], while log(FGF23) was positively associated with log[24,25(OH)_2_D/25(OH)D] and negatively with log[1,25(OH)_2_D/24,25(OH)_2_D]. Of importance, log(1 + urinary C-megalin/Cr) emerged as an independent factor positively associated with log[1,25(OH)_2_D/25(OH)D] and log[1,25(OH)_2_D/24,25(OH)_2_D].

**Table 4:** Multivariate regression analysis to elucidate factors associated with vitamin D metabolites ratio.

	log[1,25(OH)_2_D/25(OH)D]		log[24,25(OH)_2_D/25(OH)D]		log[1,25(OH)_2_D/24,25(OH)_2_D]	
	β	p	β	p	β	p
Age	0.065	0.447	0.088	0.344	−0.020	0.844
Gender, male/female 1/2	0.159	0.047*	−0.044	0.608	0.162	0.089
BMI	−0.099	0.221	−0.161	0.075	0.074	0.442
HbA1c	−0.019	0.811	0.020	0.819	−0.042	0.665
Alb	−0.185	0.036*	0.016	0.871	−0.152	0.149
log(eGFR)	0.744	<0.001*	0.561	<0.001*	0.136	0.458
log(wholePTH)	0.320	0.004*	−0.251	0.043*	0.453	<0.001*
log(FGF23)	−0.215	0.073	0.406	0.002*	−0.391	0.007*
log(1 + urinary C-megalin/Cr)	0.174	0.048*	−0.116	0.229	0.242	0.022*
R^2^ (p)	0.515 (<0.001)		0.372 (<0.001)		0.303 (<0.001)	

β is the standardized regression coefficient. *p < 0.05.

1,25(OH)_2_D = 1,25-dihydroxyvitamin D, 25(OH)D = 25-hydroxyvitamin D, 24,25(OH)_2_D = 24,25-dihydroxyvitamin D, BMI = body mass index, HbA1c = hemoglobin A1c, Alb = albumin, eGFR = estimated glomerular filtration rate, PTH = parathyroid hormone, FGF23 = fibroblast growth factor 23.

## Discussion

Results in the present study of 153 pre-dialysis CKD patients indicate that determination of urinary C-megalin excretion is clinically relevant for assessment of PTECs injury. That is based on its good correlation with urinary excretion of β_2_MG/Cr and α_1_MG/Cr (Fig. 1), and our finding that urinary loss of C-megalin might cause a reduction of serum 25(OH)D, based on its negative association with serum 25(OH)D (Table 3). Furthermore, it is likely that urinary C-megalin loss may also be involved in reductions of serum 1,25(OH)_2_D, and 24,25(OH)_2_D (Fig. 3), probably due to restricted transport of 25OH)D to PTECs mitochondria as a result of impaired megalin-mediated absorption of 25(OH)-DBP into PTECs. Additionally, our results showed that PTH and FGF23 likely retain a critical role in regulation of vitamin D metabolism from 25(OH)D to 1,25(OH)_2_D or 24,25(OH)_2_D even in the kidneys of CKD patients. When serum wholePTH findings were divided into quintiles, serum 1,25(OH)_2_D, and 24,25(OH)_2_D were shown to be decreased in the higher wholePTH quintiles, while serum 1,25(OH)_2_D/25(OH)D ratio was not significantly changed regardless of the quintile (Fig. 4). Furthermore, serum 24,25(OH)_2_D/25(OH)D ratio, but not serum 1,25(OH)_2_D/25(OH)D ratio, was not different in the various FGF23 quintiles (Fig. 5). Multivariate regression analysis findings confirmed the positive association of wholePTH with 1,25(OH)_2_D/25(OH)D and 1,25(OH)_2_D/24,25(OH)_2_D ratios, as well as the positive association of FGF23 with 24,25(OH)_2_D/25(OH)D ratio and negative association with 1,25(OH)_2_D/24,25(OH)_2_D ratio, which further confirmed the critical role of PTH and FGF23 in regulation of vitamin D metabolism even in CKD patients.

The present findings also revealed a significant and positive correlation of urinary protein with urinary C-megalin (Table 2). That finding is quite reasonable, since increased glomerular filtration of proteins, such as albumin and other low molecular weight proteins, which are taken up by PTECs *via* megalin, likely overloads the cellular endo-lysosomal system, leading to increased urinary C-megalin excretion by exocytosis from injured PTECs^10^. On the other hand, urinary C-megalin excretion, but not urinary protein excretion, was found to have a significantly negative association with serum 25(OH)D in a manner independent of eGFR, wholePTH, and FGF23 (Table 3). Interestingly, it has been speculated that renal dysfunction may accelerate vitamin D depletion^20,21^, while wholePTH and FGF23 are considered to stimulate degradation of 25(OH)D to dihydroxyvitamin D metabolites^22–25^. However, megalin-mediated protein metabolic load to PTECs caused by increased glomerular protein filtration may surpass the endocytic capacity of megalin, leading to overflow of 25(OH)D–DBP complexes into urine, possibly resulting in an association of urinary C-megalin excretion with serum 25(OH)D level. Furthermore, protein metabolic load-induced phenotypic changes in PTECs^12^ may alter intracellular trafficking of 25(OH)D to mitochondria or have an effect on its manner of activation.

It is interesting to note that serum wholePTH, but not FGF23, was associated in a significantly negative manner with serum 25(OH)D (Table 3). The present study showed that serum wholePTH associated in a negative manner with serum 25(OH)D, which was in good agreement with our previous study that serum PTH increases as serum 25(OH)D level decreases^26,27^. Therefore, reduced serum 25(OH)D due to impaired megalin-mediated absorption of 25(OH)D-DBP complexes may be responsible, at least in part for development of hyperparathyroidism as indicated by the finding of increased wholePTH, based on the suppressive effect of 25(OH)D on PTH synthesis at parathyroid gland^28^. Therefore, it is possible that urinary exosome megalin excretion has an influence to increase serum PTH, which stimulates 25(OH)D metabolism to 1,25(OH)_2_D, as compared to the effect of FGF23 to stimulate 25(OH)D metabolism to 24,25(OH)_2_D in CKD patients. Since the eGFR values of the present CKD patients were distributed between 5.0 and 58.8 mL/min/1.73 m^2^, it is reasonable to speculate that 25(OH)D metabolism is heavily affected by secondary hyperparathyroidism, based on our results indicating that serum PTH starts to increase at a level below 50 mL/min/1.73 m^2^ and then in an eGFR-dependent manner thereafter^26^. The lack of association between eGFR and 25(OH)D shown in the present study (Table 3) can be explained by a previous finding showing that vitamin D is metabolized to 25(OH)D in the liver, but not the kidneys. Therefore, the reported decrease of 25(OH)D in CKD patients may be mainly explained by increased urinary loss of 25(OH)D due to failure of megalin-mediated reabsorption of 25(OH)D, altered intracellular handling of 25(OH)D along with phenotypic changes in PTECs, or enhanced degradation of 25(OH)D to 1,25(OH)_2_D by development of secondary hyperparathyroidism^29^.

In previous studies of CKD patients with eGFR <60 mL/min/1.73 m^2^, including ours^1,19^ even though serum PTH and FGF23 were increased, either serum 1,25(OH)_2_D or 24,25(OH)_2_D was decreased along with a decline in eGFR, indicating that decreased conversion of 1,25(OH)_2_D and 24,25(OH)_2_D from 25(OH)D is the result of a reduction in functional PTECs to metabolize 25(OH)D.

However, our results showed that serum wholePTH was significantly and positively associated with log[1,25(OH)_2_D/25(OH)D] and log[1,25(OH)_2_D/24,25(OH)_2_D], and negatively associated with log[24,25(OH)_2_D/25(OH)D] (Table 4), indicating that PTH may have effects to attenuate the reduction of serum 1,25(OH)_2_D by stimulation of 1α(OH)ase and augment the reduction of serum 24,25(OH)_2_D by inhibition of 24(OH)ase. Furthermore, serum FGF23 had a positive association with log[24,25(OH)_2_D/25(OH)D] as well as a significantly negative association with log[1,25(OH)_2_D/24,25(OH)_2_D]. Together with our finding of a tendency for a negative association of serum FGF23 with log[1,25(OH)_2_D/25(OH)D], these results indicate that FGF23 may augment the reduction of serum 1,25(OH)_2_D by inhibition of 1α(OH)ase and attenuate the reduction of serum 24,25(OH)_2_D by stimulation of 24(OH)ase in CKD patients, which is in good agreement with previous findings obtained in our study of non-CKD patients^19^. The present data also indicate a positive association of urinary C-megalin excretion with log[1,25(OH)_2_D/25(OH)D] and log[1,25(OH)_2_D/24,25(OH)_2_D], which may be caused by reduced megalin-mediated uptake of 25(OH)D-DBP complexes and altered trafficking of 25(OH)D to mitochondria sites, where 1α(OH)ase and 24(OH)ase co-exist^17^, due to phenotypic changes in PTECs under conditions associated with CKD. Additionally, the Km value of 1α(OH)ase was reported to be approximately 0.1 × 10^−8^ M for 25(OH)D^30,31^, significantly lower than that of 24(OH)ase for 25(OH)D (25–5.0 × 10^−6^ M)^32,33^, showing that decreased megalin-mediated 25(OH)D reabsorption and reduced entry of 25(OH)D into mitochondrial sites might facilitate binding of 25(OH)D to 1α(OH)ase, thus increasing 1,25(OH)_2_D synthesis. Furthermore, this notion of the existence of 25(OH)D metabolism by PTH and FGF23 in CKD patient kidneys is supported by recent reports showing that FGF23 neutralization by anti-FGF23 antibody treatment significantly increased serum 1,25(OH)_2_D in CKD mice^34^ and rats^35,36^. Therefore, it is unlikely that the site of activation of 25(OH)D to 1,25(OH)_2_D might be too severely damaged to produce 1,25(OH)_2_D even in patients with CKD.

These results suggest that impaired megalin-mediated absorption of the 25(OH)D-DBP complex plays an important role in the reduction of serum 1,25(OH)_2_D and 24,25(OH)_2_D in CKD, because of their restricted absorption of 25(OH)D into PTECs, although the resultant reduction of 1,25(OH)_2_D might decrease megalin expression in PTECs^37^. On the other hand, a previous study demonstrated a severe decrease in 25(OH)D-1-hydroxylase activity in mitochondria fractions from kidney tissues obtained from CKD stage 4 patients^38^, therefore it is likely that the reduction in 25(OH)D-1α-hydroxylase and 25(OH)D-24-hydroxylase activities resulting from PTECs injury also contributes to reduced serum 1,25(OH)_2_D and 24,25(OH)_2_D in CKD patients.

The present study has several limitations. First, the number of patients examined was relatively few, mainly because we enrolled those consecutively examined at a single institution. Another limitation is that the present cross-sectional study was limited to Japanese CKD patients and it remains unclear whether the results obtained can be extended to other ethnicities. Furthermore, as shown in Fig. 2, serum 25(OH)D levels were rather low in our Japanese population, because of the relatively low level of intake of dairy products and absence of vitamin D-fortified foods. Also, it was not possible to assess causality because of the cross-sectional design. A cohort survey of disease occurrence is necessary to directly evaluate the causality of megalin and its clinical effects on vitamin D metabolites.

In summary, the present results demonstrated a significantly negative association of urinary C-megalin excretion with serum 25(OH)D, 1,25(OH)_2_D and 24,25(OH)_2_D, and positive associations with 1,25(OH)_2_D/25(OH)D and 1,25(OH)_2_D/24,25(OH)_2_D, in addition to the established roles of PTH and FGF23 in 25(OH)D metabolism, even in pre-dialysis CKD patients. Additionally, they suggest that decreased megalin-mediated reabsorption of 25(OH)D by PTECs and preferential conversion from 25(OH)D to 1,25(OH)_2_D may occur in pre-dialysis CKD patients with protein metabolic load-induced PTECs injury.

## Methods

### Ethics statement

This study was approved by the Ethics Committee of Osaka City University Graduate School of Medicine (approval #3366). All study participants provided written informed consent for sampling of blood and urine, as well as examinations of clinical records. The research was done in accordance with the Declaration of Helsinki.

### Subjects

CKD was defined by criteria proposed by the Kidney Disease: Improving Global Outcomes (KDIGO) guidelines^39^. Among CKD patients regularly followed by nephrologists at the Department of Nephrology at Osaka City University Hospital, 153 examined from April to June 2017 were enrolled in the present study. The primary conditions related to CKD in our patients were hypertensive nephrosclerosis (n = 45), diabetic nephropathy (n = 26), IgA nephropathy (n = 19), membranous nephropathy (n = 17), autosomal dominant polycystic kidney disease (n = 12), focal and segmental glomerulosclerosis (n = 5), myeloperoxidase-anti-neutrophil cytoplasmic antibody (MPO-ANCA)-associated glomerulonephritis (n = 4), membranoproliferative nephropathy (n = 2), minimal change nephrotic syndrome (n = 1), and unknown (n = 22). Patients with advanced liver disease or taking vitamin D supplements or drugs known to affect vitamin D metabolism including vitamin D derivatives were excluded.

### Measurements

Blood and urine samples were collected from all subjects in the morning after overnight fasting. Urine samples were kept on ice for 1 hour and then centrifuged at 1500 rpm for 10 minutes, as previously described^40,41^. All laboratory measurements were performed using routine assays with automated methods^19,26,42^. eGFR was calculated using the new Japanese coefficient for the abbreviated Modification of Diet in Renal Disease Study equation, including a correction factor for women of 0.739^43^. Serum calcium was corrected based on serum albumin, which was calculated as corrected calcium (cCa), as previously reported^26^. Serum wholePTH, which reacts with biologically active full-length PTH (1–84), was measured using a wholePTH assay (Scantibodies Laboratory, Inc. Santee, CA), which is a two-site immunoradiometric assay that exclusively measures PTH (1–84), with intra- and coefficients of variation (CVs) less than 2.3–6.1% and 2.9–8.9%, respectively^15,26,44,45^. Serum FGF23 was determined using a fully automated random access chemiluminescence immunoanalyzer device, the CL-JACK System [Kyowa Medex Co. Ltd., Tokyo, Japan; intra-assay CV 2.7–3.4%, inter-assay CV 1.9–6.3% (internal data)]^16,19^.

Serum levels of vitamin D metabolites were determined as noted in previous studies^1,15,26^. Briefly, serum 1,25(OH)_2_D was measured using a 1,25(OH)_2_D RIA kit (Immunodiagnostic Systems Limited, Boldon, England), and serum 25(OH)D and 24,25(OH)_2_D with a modified HPLC-tandem mass-mass spectrometry method with atmospheric pressure chemical ionization (LC-APCI-MS/MS) (HPLC System; Shimadzu, Kyoto, Japan; LC-APCI-MS/MS System; Applied Biosystems, Foster City, CA). The intra- and inter-assay CVs were 3.4–9.2% and 11.9%, respectively, for measurement of 25(OH)D, and 13.1–19.3% and 14.7%, respectively, for measurement of 24,25(OH)_2_D^1,19,46,47^.

Quantification of urinary C-megalin was performed as previously described^8,9^. Briefly, 90 μL of urine was mixed with 10 μL of a solution containing 2 mol/L Tris-HCl, 0.2 mol/L EDTA, and 10% Triton X-100 (pH 8.0), then incubated for 1 minute at room temperature, followed by reactions between the captured monoclonal antibodies immobilized on ELISA plates and the carboxy-terminal domain of megalin. An alkaline phosphatase-labeled tracer monoclonal antibody was then added to the plate and measurements were conducted using a chemiluminescent immunoassay detection system. As surrogate markers of renal tubule injury, urinary concentrations of Cr, β_2_MG, and α_1_MG were determined using an automated instrument (7170 S; Hitachi High-Technologies Corp., Tokyo, Japan), with CRE-S (Denka Seiken Co., Ltd.), BMG-Latex (Denka Seiken Co., Ltd.), and αMi-Latex (Denka Seiken Co., Ltd.) kits, respectively, as previously described^8,9^. The urinary concentration of each marker was normalized to that of Cr, then expressed as g/g Cr (protein), µg/g Cr (β_2_MG), mg/g Cr (α_1_MG), and pmol/g Cr (C-megalin).

### Statistical analysis

Continuous variables with normal distribution are expressed as the mean ± SD. Median (range) values were used for continuous variables with skewed distribution. Simple regression analysis was performed using a non-parametric Spearman’s rank correlation test. Multiple regression analyses were performed after logarithmic transformation of eGFR, wholePTH, FGF23, urinary protein/Cr, urinary C-megalin/Cr, urinary β_2_MG/Cr, urinary α_1_MG/Cr, serum 25(OH)D, 1,25(OH)_2_D/25(OH)D ratio, 24,25(OH)_2_D/25(OH)D ratio, and 1,25(OH)_2_D/24,25(OH)_2_D ratio, because of their transformation to an approximated normal distribution. Comparison of two regression slopes of 1,25(OH)_2_D and 24,25(OH)_2_D was performed as described previously^48,49^. Association between urinary C-megalin and quintiles of β_2_MG or α_1_MG values were analyzed by one-way ANOVA (analysis of variance), followed by Dunnett’s test. Also, association between serum vitamin D metabolites and quintiles of wholePTH or FGF23 values were analyzed by one-way ANOVA (analysis of variance), followed by Dunnett’s test. All statistical analyses were performed using the Stat View V system (Abacus Concepts, Berkeley CA) and JMP Pro 12 (SAS Corporation, Cary, North Carolina, United States) on a Windows computer. P values < 0.05 were considered to indicate statistical significance.
